# Randomised Controlled Feasibility Trial of Face‐To‐Face Diabetes Self‐Management Education Shows High Completion Rates Are Needed to Improve Patient‐Reported Outcomes

**DOI:** 10.1111/dom.70917

**Published:** 2026-05-27

**Authors:** Gemma A. Lewis, David M. Hughes, Greg J. Irving, John P. Wilding, Kevin J. Hardy

**Affiliations:** ^1^ Department of Cardiovascular and Metabolic Medicine Institute of Life Course and Medical Sciences, University of Liverpool Liverpool UK; ^2^ Department of Diabetes and Endocrinology St Helens Hospital, Mersey and West Lancashire Teaching Hospitals NHS Trust St Helens UK; ^3^ Department of Health Data Science, Institute of Population Health University of Liverpool Liverpool UK; ^4^ Health Research Institute Edge Hill University Omskirk UK

**Keywords:** clinical trial, dose–response relationship, effectiveness, patient‐reported outcomes, randomised trial, type 2 diabetes

## Abstract

**Aims:**

To examine the dose–response relationship between diabetes self‐management education (DSME) attendance and psychological outcomes in type 2 diabetes, assessing whether minimal attendance (10%) produces clinically meaningful improvements and comparing outcomes at the internationally adopted 60% completion benchmark with full (100%) completion.

**Materials and Methods:**

This randomised feasibility trial enrolled 120 adults (≥ 18 years) due to attend a UK DSME programme. Participants were randomised to receive 100% (routine DSME), 60%, 10% or 0% (delayed DSME). Primary outcomes were changes in self‐management skills; secondary outcomes included health‐related quality of life (HRQoL) and diabetes distress.

**Results:**

Participants had a mean age of 61 years, mean HbA_1c_ of 8.4% (68 mmol/mol) and median diabetes duration of 8 years. Significant between‐group differences were observed in change scores for self‐management skills (*F*[3,109] = 6.914, *p* < 0.001) and diabetes distress (*F*[3,108] = 7.369, *p* < 0.001). Education dose explained 16%–17% of the variance demonstrating a dose–response relationship (η*p* ^2^ = 0.16–0.17) with a moderate effect size (*n* ^2^ = 0.16, 95% CI [0.04, 0.27]). Within‐group statistical improvements were observed for both 100% and 60% completion, but clinically meaningful improvements across all psychological domains occurred only with full (100%) completion. No significant between‐group differences were observed for HRQoL.

**Conclusions:**

In this randomised feasibility trial, clinically significant psychological benefits from DSME required full (100%) programme completion. Attendance thresholds of ≤ 10% or partial completion (≥ 60%) did not yield meaningful improvements in diabetes distress or HRQoL. These findings suggest that existing performance indicators based on partial attendance may not reflect meaningful benefits and support 100% DSME attendance as the gold standard.

**Trial Registration:**

Clinicaltrials.gov identifier: NCT06419907

## 1 Introduction

Diabetes is a complex condition leading to significant disability and premature mortality affecting an estimated 589 million people worldwide [1]. In the United States, approximately 12% of the population is affected, representing one of the highest national prevalences globally and the greatest diabetes‐related healthcare expenditure [1, 2]. Type 2 diabetes accounts for 90% of cases, with prevalence disproportionately higher among individuals experiencing socioeconomic deprivation [3].

Diabetes self‐management education (DSME) is internationally recommended for all individuals newly diagnosed with type 2 diabetes and plays a critical role in improving glycaemic control, preventing microvascular and macrovascular complications [4, 5, 6] and is associated with a ~45% reduction in mortality risk [7]. Despite broad consensus on its efficacy, global attendance and completion rates remain low: in the UK, only 9% start DSME [8] and as few as 1.1% complete it [9], while in the US fewer than 7% attend within the first year of diagnosis [10].

Both NHS England and the Centres for Disease Control and Prevention define DSME ‘attendance’ as ≥ 10% engagement and ‘completion’ as ≥ 60%; however, these thresholds are arbitrary and not based on empirical evidence defining an effective educational dose [11, 12]. Existing evidence predominantly reflects outcomes among programme completers [13, 14, 15, 16, 17] or uses intention to treat analyses, which may attenuate the observed effects when intervention adherence is poor [18]. Consequently, whether partial participation confers meaningful clinical or psychosocial benefit remains unclear [18, 19].

Whether a dose–response relationship exists between DSME exposure and outcomes, particularly patient‐reported outcome measures (PROMs), remains unknown. We previously identified that full programme completion improves self‐management skills and short‐term HRQoL, but no studies reported outcomes for partial completion, and the long‐term impact on HRQoL and diabetes distress remains uncertain [18]. Addressing this gap has important implications for DSME delivery and service‐level guidance, including programme evaluation, and provides the rationale for evaluating the effects of varying levels of DSME participation.

This study aimed to investigate the dose–response relationship between DSME attendance and self‐management skills, diabetes distress and HRQoL outcomes in adults with type 2 diabetes.

## 2 Materials and Methods

### 2.1 Study Design

Our study was a single‐centre, four‐arm randomised feasibility trial conducted between May 2024 and February 2025 at a large English teaching NHS hospital providing specialist diabetes care. The study was designed, conducted, analysed and reported in accordance with the consolidation standards of reporting trials (CONSORT) statement for pilot and feasibility trials [20]. Study protocol has been published previously [21], and ethical approval was obtained from the London—Surrey Borders Research Ethics Committee (24/LO/0235) on 22 April 2024, prior to trial registration with Clinicaltrials.gov (NCT06419907). All participants provided written informed consent prior to enrolment in the study. This research was conducted ethically in accordance with the World Medical Association Declaration of Helsinki.

### 2.2 Participants

Eligible participants were adults aged ≥ 18 years with a recorded clinical diagnosis of type 2 diabetes who met national eligibility criteria for DSME, could provide informed consent and were responsible for daily management of their diabetes were invited to participate. We excluded individuals with type 1, type 3c, monogenic or gestational diabetes; those who were unable to attend group‐based education; or had received structured education in the 12 months before recruitment.

Participants were identified from multiple complementary streams to achieve a sample representative of the local population, rather than just those requiring specialist care: [1] direct referrals for DSME from primary care settings; [2] via primary care reports of newly coded type 2 diabetes patients; [3] eligible patients identified in secondary care clinics. Clinical care was not altered by study participation.

### 2.3 Intervention

Participants (*n* = 120) were randomised to one of four groups using a permuted block strategy with block sizes of 4, 8 or 12, implemented via an online randomisation platform designed for clinical trials [22]. The four groups were defined by the proportion of DSME received:

- 100% group: full DSME programme (6 h)
- 60% group: partial DSME (3 h 36 min)
- 10% group: minimal DSME (36 min)
- 0% group: delayed DSME

Due to the nature of the study design, researcher and participant blinding was not feasible. Participants were informed of potential disadvantages, including being allocated to a group receiving a reduced or delayed DSME dose during the study.

Participants in the 100%, 60% and 10% groups attended the same DSME programme provided to routine clinical patients, with typically 10–15 patients per group. Group DSME was delivered weekly with routine patients attending a single 6‐h session. For research participants, DSME was scheduled within 3 weeks of baseline assessment. Participants randomised to the 0% group did not attend DSME as part of the trial. Participants were asked not to access DSME programmes elsewhere during the study period.

Programme content was benchmarked against national programmes and is accredited by the UK national quality institute for self‐management education & training (QISMET) [23]. While the amount of DSME received differed between‐groups, the core content and underpinning adult learning principles were consistent, guided by a structured lesson plan. This approach ensured participants were typically exposed to similar information at the same point in each session; for example, all participants received identical content during the first 36 min (10% of DSME), regardless of whether they attended only part of the session or completed the full programme (Table 1). At the end of the study, participants in 60%, 10%, and 0% groups were offered the full DSME programme.

Participants randomised to the delayed group completed PROMs at baseline and at 4 months but did not attend DSME during the study period.

### 2.4 Data Collection

Following written informed consent, baseline demographic and clinical data were collected, including age, sex, diabetes duration, latest HbA_1c_, Index of Multiple Deprivation (IMD) decile as a proxy for socioeconomic status and systemic disadvantage [24] and Charlson Comorbidity Index (CCI) as a measure of comorbidity burden and prognostic risk [25, 26]. Data were obtained from the patient electronic record system and recorded using REDCap, a secure data capture platform for clinical research studies [27, 28].

### 2.5 Endpoints and Assessments

As a feasibility trial, the primary outcome related to the practicality of delivering DSME at varying lengths, along with refinement of data collection procedures and assessment of intervention fidelity and adherence.

Pre‐specified primary clinical outcome was change in self‐management activities, assessed using the diabetes self‐management questionnaire (DSMQ) revised scale [29]. Secondary clinical endpoints included changes in diabetes distress, measured using problem areas in diabetes (PAID) [30] and HRQoL, measured using the PROMIS global health V1.2 scale [31]. Validated questionnaires were completed at baseline and 3–4 months post‐intervention. This follow‐up interval was selected to allow sufficient time for behavioural change while minimising the risk of not receiving the full DSME programme as recommended by international guidelines [5, 6].

Landmark trials such as DESMOND [13] frequently use HbA_1c_ as a primary endpoint, and despite being powered to detect changes in HbA_1c_, often found no significant improvement 4 months post‐intervention. This study was not powered to detect changes in HbA_1c_, and given the behavioural focus of DSME, PROMs were prioritised as primary outcomes. This approach is consistent with evidence suggesting DSME primarily influences behavioural change to support glycaemic control; efficacy measures should therefore focus on behavioural and psychosocial outcomes [4]. As a feasibility study, this trial was not powered for hypothesis testing but to estimate effect sizes for a future definitive RCT; sample sizes of 24–50 participants per group are considered sufficient [32]. Allowing for an anticipated attrition rate of approximately 25% resulted in a sample of 120 participants across the four arms.

Minimal clinically important differences (MCIDs) were used to assess clinical relevance. The MCIDs were defined as > 2 T‐score points for PROMIS global health, > 0.6 for DSMQ and > 6.48 for PAID (calculations provided in Tables S2 and S3).

### 2.6 Statistical Analysis

Continuous data are presented as mean ± standard deviation (SD) and categorical data as frequencies and percentages. Normality was assessed using the Shapiro–Wilk test and visual inspection of histograms and Q‐Q plots. PROMs were treated as continuous variables. Within‐group pre‐ to post‐intervention changes were analysed using two‐tailed paired *t*‐tests for parametric data and Wilcoxon signed ranks test for non‐parametric data. Between‐group differences across the four DSME exposure levels were assessed using a one‐way ANOVA model, with ANCOVA applied where appropriate to adjust for baseline values. Post hoc Tukey and Bonferroni‐adjusted tests were conducted where significant between‐group comparisons were identified. Additional correlation analyses were performed to explore the relationship between diabetes duration, socioeconomic deprivation, CCI and endpoints. Statistical significance was defined as *α* = 0.05. All analyses were conducted using IBM SPSS Premium 29.

### 2.7 Data and Resource Availability

De‐identified datasets arising from this study will be made available upon reasonable request addressed to the corresponding author and with ethical approval.

## 3 Results

### 3.1 Participant Recruitment

A total of 712 individuals were screened for eligibility of whom 370 (52%) were excluded. Reasons for exclusion included type 1 or type 3c diabetes (*n* = 160, 43%), prior attendance at structured education within the last 12 months (*n* = 77, 21%), ineligible for group education (*n* = 40, 11%), declining DSME (*n* = 43, 12%) and other reasons (*n* = 50, 14%). Of the remaining 342 eligible individuals, 139 provided consent and 120 were randomised and completed the assigned intervention achieving target group sizes (*n* = 30 per arm).

**FIGURE 1:**
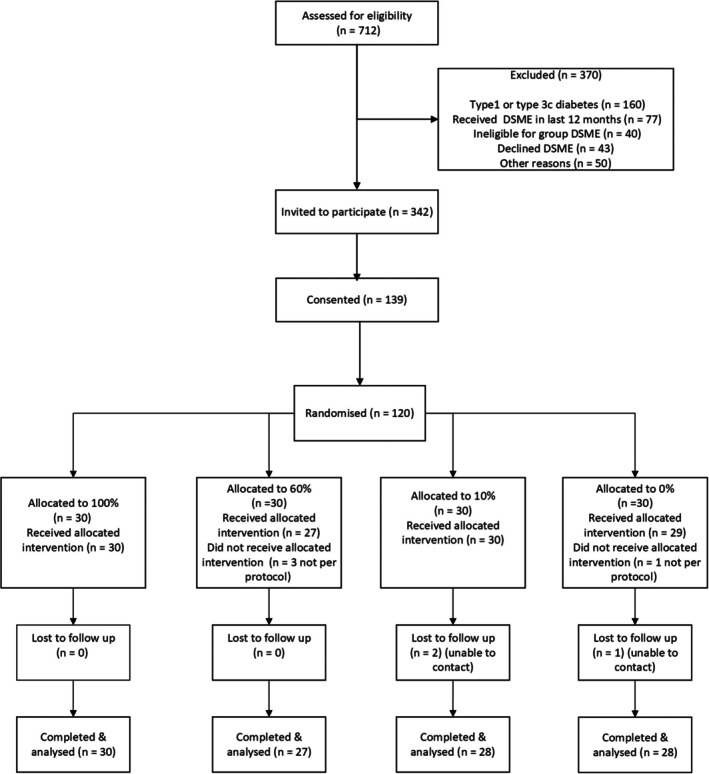
CONSORT flowchart.

Most participants were recruited via specialist clinics (65%), with lower recruitment from primary care referrals (26%) and newly diagnosed primary care reports (9%) (Table S4). Overall, recruitment exceeded initial timelines. Participants' flow through the trial is demonstrated in the CONSORT [20] flowchart (Figure 1).

### 3.2 Adherence and Feasibility

Overall retention was high, with 94% of participants completing the study. Delivery of DSME alongside routine patients proved feasible and enabled flexible scheduling; however, some participants requested sessions to be rescheduled ≥ 3 times.

Adherence was assessed through reviewing the number of dropouts and non‐adherence to the randomised intervention. Four participants were excluded from analysis due to protocol deviations: three attended insulin safe start education [23] during the trial to meet their medical needs and one completed 100% DSME instead of the randomised 60%. An additional three participants were lost to follow up. These cases were excluded because the study is specifically examining differing DSME completion levels so an intention to treat analysis was not appropriate.

Eight serious adverse events were reported in four participants (three in the 100% group and one in the 0% group). None were deemed to be study‐related and all represented exacerbations of pre‐existing health conditions. No concerns were reported with the randomisation process.

### 3.3 Baseline Characteristics

Participants had median age 61 years (IQR 14), elevated HbA_1c_ levels of 8.4% (68 mmol/mol), moderate comorbidity burden and a median diabetes duration of 8 years (IQR 16) and were broadly comparable across the randomised groups as shown in Table 1. All 120 randomised participants were of white ethnicity, reflective of the local population. Participants were from some of the most deprived neighbourhoods in the UK. A clinically relevant difference in HbA_1c_ was observed, ranging from 8.7% (71 mmol/mol) in the 60% DSME group to 8.0% (64 mmol/mol) in the 0% group. HbA_1c_ results were available for all secondary care participants at the time of recruitment and were obtained from the electronic medical record (within 3 months of recruitment) for those who were recruited via primary care referrals or newly diagnosed primary care reports.

**TABLE 1:** Participant baseline measures.

Characteristics	All groups (*n* = 113)	100% (*n* = 30)	60% (*n* = 27)	10% (*n* = 28)	0% (*n* = 28)	*p*
% specialist clinic	65%	67%	59%	64%	68%	0.77
Age (years)	61 (14)	60 (13)	62 (12)	62 (17)	60 (16)	0.54
% female	50%	57%	52%	32%	57%	0.20
IMD	3 (4)	3 (5)	3 (4)	2 (4)	3 (4)	0.45
Diabetes duration (years)	8 (16)	9 (14.3)	7 (12.6)	7 (17.4)	9 (16.4)	0.67
HbA_1c_ %	8.4 (5.0)	8.2 (5.7)	8.7 (4.3)	8.6 (5.2)	8.0 (4.8)	0.49
CCI	3 (2)	3 (2)	3 (2)	3 (2)	3 (4)	0.72
Baseline DSMQ	5.66 ± 1.48	5.56 ± 1.16	5.51 ± 1.30	5.93 ± 1.29	5.65 ± 1.69	0.72
Baseline PAID	22.50 (28.75)	30.00 (39.06)	21.25 (18.75)	10.00 (21.88)	23.75 (30.63)	0.03
Baseline PROMIS mental health	42 (15.55)	42 (10.93)	44 (13.70)	44 (18.58)	38 (18.58)	0.24
Baseline PROMIS physical health	37 ± 9.30	38 ± 9.42	38 ± 7.89	40 ± 8.70	34 ± 10.63	0.20

*Note:* Non‐normally distributed data reported as median (IQR); normally distributed data reported as mean ± SD.

Abbreviations: CCI, Charlson Comorbidity Index; DSMQ, diabetes self‐management questionnaire; IMD, Index of Multiple Deprivation; PAID, problem areas in diabetes; PROMIS, patient‐reported outcomes measurement information system.

**FIGURE 2:**
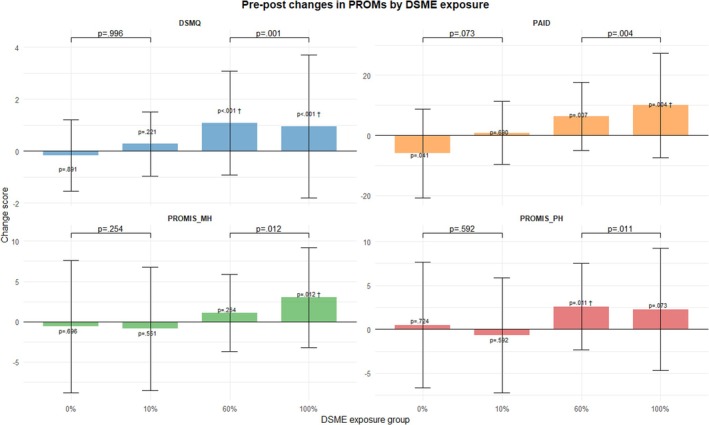
Patient‐reported outcome measure change scores by DSME exposure group. Pre–post changes in patient‐reported outcome measures (PROMs) by level of DSME exposure. Bars represent mean change scores from baseline to follow‐up for four groups receiving 0%, 10%, 60%, or 100% of the DSME programme. Error bars indicate standard deviation. *p* values shown above each bar represent within‐group pre–post change significance (Wilcoxon signed‐rank test for DSMQ; paired *t*‐tests for PAID, PROMIS MH, and PROMIS PH). Between‐group comparisons were analysed using ANOVA or ANCOVA as appropriate (see Table 2). † Indicates changes meeting or exceeding the minimal clinically important difference (MCID) for that measure. PAID scores reflect diabetes‐related distress (higher scores indicate greater distress), while higher scores on DSMQ and PROMIS scales indicate better self‐management and HRQoL, respectively.

A difference in baseline diabetes distress was observed (*p* = 0.029), with higher scores in the 100% group; mean scores ranged from 18.4 in the 10% group to 32.9 in the 100% group. Clinical outcomes for this study, however, were based on mean change between‐groups for PROMs, not pre‐ and post‐score results reducing the impact of baseline differences between study groups (PROM change scores are presented in Figure 2 and Table S5). No significant differences were seen in baseline DSMQ, PROMIS global mental health (MH) and physical health (PH) scores. Group 0% had MH and PH T‐Point scores six points lower than other groups. Baseline PROMIS T‐scores for both MH and PH were below the population mean of 50 across all groups [31, 33].

### 3.4 Self‐Management (DSMQ)

A significant between‐group difference in self‐care skills was observed using one‐way ANOVA (*F*[3,109] = 6.914, *p* < 0.001; *η* ^2^ = 0.160, 95% CI [0.040, 0.267]) as demonstrated in Table 2, indicating that 16% of the variance in DSMQ change was attributable to DSME dose. Tukey post hoc comparisons showed that participants completing 100% and 60% of DSME demonstrated significantly greater improvements than those attending 10% (*p* < 0.05) and 0% (*p* < 0.01). No differences were observed between 100% and 60% (*p* = 0.996) or between 10% and 0% (*p* = 0.917). Both the 100% and 60% groups exceeded the DSMQ MCID, indicating clinically meaningful improvements.

**TABLE 2:** Between‐group comparison of PROM change scores.

Outcome	Group	Mean change ± SD/median (IQR)	*F*/ANCOVA *F*	*p*	Partial *η* ^2^	MCID exceeded?
DSMQ	100%	0.96 (2.75)	6.914	< 0.001	0.160	Yes
	60%	1.08 (2.0)				Yes
	10%	0.28 (1.24)				No
	0%	−0.17 (1.38)				No
PAID	100%	8.14 ± 2.44	7.369 (ANCOVA)	< 0.001	0.170	Yes
	60%	7.14 ± 2.53				Yes
	10%	2.54 ± 2.52				No
	0%	−6.51 ± 2.48				No
PROMIS MH	100%	3.0 ± 6.18	2.021	0.115	—	Yes
	60%	1.07 ± 4.77				No
	10%	−0.88 ± 7.67				No
	0%	−0.61 ± 8.22				No
PROMIS PH	100%	2.27 ± 6.95	1.574	0.200	—	Yes
	60%	2.58 ± 4.92				Yes
	10%	−0.67 ± 6.56				No
	0%	0.48 ± 7.15				No

*Note:* DSMQ change scores reported as median (IQR); PAID and PROMIS change scores as mean ± SD. MCID thresholds: DSMQ > 0.6; PAID > 6.48; PROMIS MH/PH > 2 T‐score points. ANCOVA used for PAID change scores to adjust for baseline differences.

### 3.5 HRQoL (PROMIS)

Despite significant pre–post changes within individual groups, no statistically significant differences were found between‐groups for HRQoL, as measured by PROMIS MH (*F*[3,109] = 2.021, *p* = 0.115) or PROMIS PH (*F*[3,109] = 1.574, *p* = 0.200). Nevertheless, within‐group analysis indicated that both the 100% and 60% DSME groups exceeded the MCID for PH, while only the 100% completion group exceeded the MCID for MH. These findings suggest clinically meaningful improvements in HRQoL may occur despite the absence of statistically significant between‐group differences.

### 3.6 Diabetes Distress (PAID)

After adjustment for baseline values, a significant group effect was observed for change in diabetes distress (F[3,108] = 7.369, *p* < 0.001; ηp^2^ = 0.170, 95% CI [0.07, 0.27]), with 17% of the variance attributable to DSME dose. Baseline distress was also a significant predictor of change (ηp^2^ = 0.116, 95% CI [0.03, 0.20]). Mean reductions in diabetes distress were highest in the 100% group (8.1 ± 2.44), followed by the 60% group (7.1 ± 2.53). The 10% group showed smaller reductions (2.5 ± 2.52), whereas the 0% group demonstrated an increase in distress (−6.5 ± 2.48). Bonferroni‐adjusted post hoc comparisons showed significant differences between 100% versus 0% (mean difference = 14.7, 95% CI [5.35, 23.94], *p* < 0.001) and 60% versus 0% (mean difference = 13.6, 95% CI [4.11, 23.18], *p* < 0.001), with no significant differences between adjacent dose groups (Table S6).

A general linear model demonstrated a significant interaction between DSME dose and CCI on self‐management skill change (F[3,97] = 5.971, *p* < 0.001), whereas no significant interactions were observed for PAID or PROMIS outcomes. This suggests that the impact of comorbidity on self‐care skills varies depending on the amount of education received.

## 4 Discussion

Type 2 diabetes substantially increases morbidity and premature mortality and structured DSME is widely accepted as fundamental for effective diabetes management. Despite this, uncertainty remains regarding the minimum level of engagement required to achieve meaningful psychological benefits, and current attendance benchmarks are not evidence‐based.

This randomised feasibility trial demonstrates a clear dose–response relationship between DSME attendance and patient‐reported outcomes. Increasing levels of attendance were associated with progressively greater improvements in self‐management skills and psychological well‐being. Participants completing 60% or 100% of DSME achieved significant improvements in self‐management skills, both exceeding MCIDs. But only full completion (100%) produced clinically meaningful reductions in diabetes‐related distress and improvements in mental HRQoL. By contrast, minimal attendance (≤ 10%) offered no measurable benefit, while non‐attendance (0%) was associated with deterioration in self‐management skills, reduced quality of life and increased distress, even among participants motivated to engage with behavioural change (*p* = 0.041), underscoring the importance of timely referral and participation in DSME.

These findings suggest that DSME effects are not uniform across PROMs and may instead reflect distinct dose thresholds for behavioural and psychological change. Early exposure to DSME may be sufficient to initiate improvements in self‐management behaviours, whereas psychological outcomes such as reduced diabetes distress may require sustained engagement, reinforcement of learning and repeated cognitive and emotional processing over the full course of the programme. This pattern suggests that psychological benefits may only emerge once a minimum threshold of DSME exposure is reached, rather than increasing in a linear fashion with greater attendance.

Health‐related quality of life outcomes further support this distinction. Improvements to physical HRQoL were observed at 60% completion, whereas improvements to mental HRQoL were restricted to full completion. This divergence may reflect the sequencing and timing of DSME content delivery, where practical behavioural components such as chair‐based exercises are delivered earlier, potentially amplifying gains in physical functioning. In contrast, improvements to mental HRQoL may require sustained engagement with later‐stage content, including problem‐solving, peer discussion and cognitive reframing of long‐term condition management. This interpretation is consistent with prior RCTs reporting that DSME can improve some aspects of quality of life, although effects are often heterogeneous and may diminish over time or be limited to specific subscales of health status measures [18, 19, 34, 35].

Collectively, these findings suggest that the impact of DSME on quality of life may depend not only on total exposure but also on the sequencing and reinforcement of content across the programme. These observations highlight the potential importance of programme structure in optimising patient‐reported outcomes, particularly for mental HRQoL and support further evaluation of DSME design and delivery models.

From a service delivery perspective, these findings suggest that current DSME performance indicators based on ≥ 60% attendance may adequately capture behavioural improvement but may underestimate the level of engagement required for psychological benefit. This raises the possibility that existing audit frameworks do not fully reflect patient‐centred outcomes through DSME.

While the national diabetes audit (NDA) and quality and outcomes framework (QOF) already capture key indicators for DSME referral and attendance, integration of completion metrics could support more nuanced evaluation of programme effectiveness, distinguishing between behavioural and psychological outcomes, thereby enhancing accountability and interpretation of service‐level performance.

This study has several limitations. As a feasibility trial, it was not powered to detect small between‐group differences or to confirm causal mechanisms underlying observed effects. The sample was predominantly white and recruited largely from specialist clinics, limiting generalisability to more diverse and primary care‐based populations. In addition, recruitment from primary care was less effective than anticipated due to challenges in contacting eligible patients and low interest in DSME, consistent with national trends [8]. Recruitment through specialist clinics proved more efficient but may limit scalability in a larger trial and could result in a sample that is limited to patients with more severe or complex cases of diabetes.

Participants had a median diabetes duration of 8 years, which may limit the applicability of findings to newly diagnosed patients, who are the primary target of structured DSME programmes [5]. Finally, while randomisation was robust, the need for DSME sessions to be rescheduled on multiple occasions for some participants illustrates logistical challenges for scaling DSME delivery. Recruitment was also temporarily reduced by a local hospital freeze on routine DSME during the first 2 months of the study to accommodate electronic health record changes, although this did not impede overall feasibility. Despite these limitations, retention was high (94%), demonstrating the study's feasibility, with flexible scheduling supporting programme engagement. Recruitment patterns suggest engagement was most effective through specialist clinics, perhaps reflecting the value of face‐to‐face discussions with specialists to facilitate understanding of the benefits and importance of DSME attendance. However, this highlights the potential barriers to engagement in primary care and underscores the need for improved recruitment strategies in future studies.

Future studies should explore strategies to enhance recruitment from primary care, increase ethnic diversity and investigate how session sequencing, delivery models and participant characteristics influence engagement and outcomes. Tools that leverage electronic health records for cohort discovery and recruitment, such as the UK FARSITE system [36] and comparable US distributed data networks (e.g., PCORnet) may facilitate more efficient and representative recruitment across primary care populations, thereby enhancing the generalisability of study findings.

In conclusion, this study suggests a dose–response relationship between DSME attendance and patient‐reported outcomes in type 2 diabetes, with different thresholds for behavioural and psychological benefits. While 60% attendance appears sufficient to improve self‐management skills, full programme completion may be required for optimal psychological outcomes. Timely access to DSME and full programme participation appear important for optimising patient outcomes in type 2 diabetes. These findings suggest that DSME performance metrics could be refined to reflect differential effects across outcomes, with partial attendance capturing behavioural change and full completion reflecting psychological benefit. From a service delivery and policy perspective, current international benchmarks may underestimate the level of engagement required for optimal psychological outcomes. This feasibility trial provides preliminary evidence to inform the development of more nuanced monitoring frameworks for DSME delivery and evaluation in routine clinical practice.

## Author Contributions

G.A.L. conceptualised the study. G.A.L., G.J.I., J.P.W. and K.J.H. contributed to study design. G.A.L. was responsible for data collection and analysis. K.J.H. and J.P.W. supervised the study. G.A.L. wrote the first draft of the manuscript. D.M.H. provided statistical analysis guidance. D.M.H., G.J.I., J.P.W. and K.J.H. contributed to the discussion and edited the manuscript. All authors approved the final version of the manuscript. G.A.L. is the guarantor of this work and, as such, had full access to all the data in the study and takes responsibility for the integrity of the data and the accuracy of the data analysis.

## Funding

The authors have nothing to report.

## Conflicts of Interest

G.J.I. is the National NIHR Research Delivery Network lead for General Practice. J.P.W. reports consultancy/advisory board work for the pharmaceutical industry contracted via the University of Liverpool in the last 36 months (no personal payment) for Alnylam, Amgen, AstraZeneca, Boehringer Ingelheim, Cytoki, Kailera, Lilly, Menarini, Metsera, Napp, Novo Nordisk, Pfizer, Prosciento, Response Pharmaceuticals, Rhythm Pharmaceuticals, Saniona, Shionogi and Ysopia; funding for clinical trials from Amgen, AstraZeneca and Novo Nordisk and personal honoraria/lecture fees from AstraZeneca, Boehringer Ingelheim, Medscape, Novo Nordisk and Menarini. J.P.W. is past president of the World Obesity Federation, a member of the Association for the Study of Obesity, Diabetes UK, EASD, ADA, Society for Endocrinology and the Rank Prize Funds Nutrition Committee. From 2009 to 2024 he was national lead for the Metabolic and Endocrine Speciality Group of the UK NIHR Clinical ResearchNetwork. G.A.L., D.M.H. and K.J.H. report no conflicts of interest.

## Supporting information


**Table S1:** DSME core content.
**Table S2:** Minimal clinically important difference (MCID) calculations for PAID.
**Table S3:** Minimal clinically important difference (MCID) calculations for DSMQ.
**Table S4:** Source of randomised participants.
**Table S5:** Patient‐reported outcome change scores.
**Table S6:** ANCOVA pairwise comparisons for PAID change scores.

**Data S1:** CONSORT 2010 checklist.

## Data Availability

The data that support the findings of this study are available from the corresponding author upon reasonable request.
