# Supplementary material for: Randomised Controlled Feasibility Trial of Face‐To‐Face Diabetes Self‐Management Education Shows High Completion Rates Are Needed to Improve Patient‐Reported Outcomes
Source: Diabetes Obes Metab. 2026 May 27;28(8):6997–7005. doi: 10.1111/dom.70917 (PMC13341371; doi:10.1111/dom.70917)
Supplement: Supplementary file 1 — Table S1: DSME core content. Table S2: Minimal clinically important difference (MCID) calculations for PAID. Table S3: Minimal clinically important difference (MCID) calculations for DSMQ. Table S4: Source of randomised participants. Table S5: Patient‐reported outcome change scores. Table S6: ANCOVA pairwise comparisons for PAID change scores. [file DOM-28-6997-s001.docx]

Supplementary Table 1: DSME core content

| **Group (%)** | | | **Content** |
| --- | --- | --- | --- |
| 10. | 60% | 100% | What is type 2 diabetes including symptoms and etiology |
|  |  |  | Fears and worries |
|  |  |  | Monitoring diabetes and blood glucose levels |
|  |  |  | Hypoglycemia |
|  |  |  | Hyperglycemia |
|  |  |  | Complications associated with diabetes including neuropathy, nephropathy, retinopathy, macrovascular risks. |
|  |  |  | Preventing complications including foot and eye screening |
|  |  |  | Weight management, healthy eating including food labels. |
|  |  |  | Physical activity / exercise |
|  |  |  | Alcohol and smoking |
|  |  |  | Medication |
|  |  |  | Mental health and stress |
|  |  |  | Sick days |
|  |  |  | Driving |
|  |  |  | Technology |
|  |  |  | Travel |
|  |  |  | Eating out |
|  |  |  | What care should you receive / annual review / blood tests |
|  |  |  | Working with your healthcare team and taking ownership |

*At the end of the study participants in groups 60%, 10% and 0% were offered a full DSME programme.*

# Supplementary Table 2: Minimal clinically important difference (MCID) calculations for PAID

| **Author** | **Mean baseline score** | **Participants (type 2 diabetes only)** |
| --- | --- | --- |
| Sturt, 2008(1) | 21 | 225 |
| De Brito, 2016(2) | 45.63 | 37 |
|  | 56.18 | 74 |
| Kellow 2019(3) | 21 | 34 |
| Reaney, 2013(4) | 18 | 371 Germany |
|  | 35 | 148 Spain |
|  | 36.3 | 162 Spain |
| Welch 1997 | 29.2 | 256 |
| Schmitt 2015(5) | 36 | 206 |
| Polonsky, 1995(6) | 54.5 | 451 |
| Sigurdardottir, 2008(7) | 27.88 | 84 |
| Reddy 2013(8) | 26.32 | 184 |
| Delahanty, 2007(9) | 24.6 | 212 insulin |
|  | 14.7 | 221 diet only |
|  | 17.8 | 382 oral agent |
| **Mean PAID score** | **30.94** | |
| **sd** | **12.97** | |
| **sd^2^** | **6.48** | |

PAID MCID ≥6.48

Supplementary Table 3: Minimal clinically important difference (MCID) calculations for DSMQ

| **Author** | **Mean baseline score** | **Participants (type 2 diabetes only)** |
| --- | --- | --- |
| Schmitt, 2013(10) | 6.6 | 111 |
| Schmitt, 2018 (11) | 6.4 | 255 |
| Schmitt, 2017 (12) | 5.4 | 67 |
| Schmitt, 2018 (13) | 6.3 | 107 |
| Schmitt, 2020 (14) | 5.4 | 180 |
| Bukhsh, 2022(15) | 5.92 | 75 |
| Eroglu, 2021(16) | 3.21 | 80 |
| Vincze, 2020 (17) | 7.65 | 221 |
| Popoviciu, 2022(18) | 6.32 | 159 |
| Oliveira, 2024(19) | 6.5 | 365 |
| **Mean DSMQ score** | **5.85** | |
| **sd** | **1.09** | |
| **sd^2^** | **0.55** | |

DSMQ MCID ≥0.55

Supplementary Table 4: Source of randomised participants

| **Source of recruitment** | **Study group, n (%)** | | | | **Total, n (%)** |
| --- | --- | --- | --- | --- | --- |
|  | **100%** | **60%** | **10%** | **0%** |  |
| Specialty clinics | 20 (67) | 19 (63) | 20 (67) | 19 (63) | 78 (65) |
| Primary care referrals | 8 (27) | 6 (20) | 7 (23) | 10 (33) | 31 (26) |
| Newly diagnosed primary care report | 2 (6) | 5 (17) | 3 (9) | 1 (3) | 11 (9) |

Supplementary Table 5: Patient reported outcome change scores

|  | 100% | 60% | 10% | 0% |
| --- | --- | --- | --- | --- |
| n | 30 | 27 | 28 | 28 |
| DSMQ change | .96 (2.75) Ϯ | 1.08 (2.0) Ϯ | .28 (1.24) | -.17 (1.38) |
| P (pre & post) | <.001** | <.001** | .221 | .891 |
| PAID change | 9.96±17.34 Ϯ | 6.39 ±11.34 | .80±10.54 | -5.98±14.76 |
| P (pre & post) | .004** | .007** | .690 | .041 |
| PROMIS MH change | 3.0±6.18 Ϯ | 1.07 ±4.77 | -.88±7.67 | -.61±8.22 |
| P (pre & post) | .012* | .254 | .551 | .696 |
| PROMIS PH change | 2.27±6.95 Ϯ | 2.58 ± 4.92 Ϯ | -.67±6.56 | .48±7.15 |
| P (pre & post) | .073 | .011* | .592 | .724 |

Nonparametric data reported as median (IQR), parametric data reported as mean ±SD. Ϯ ≥ calculated MCID score.

Supplementary Table 6: ANCOVA pairwise comparisons for PAID change scores

| (I) Group | (J) Group | Mean Difference (I-J) | Std. Error | Sig.^b^ | 95% Confidence Interval for Difference^b^ | |
| --- | --- | --- | --- | --- | --- | --- |
|  |  |  |  |  | Lower Bound | Upper Bound |
| 100% | 60% | 1.000 | 3.539 | 1.000 | -8.513 | 10.513 |
|  | 10% | 5.603 | 3.567 | .715 | -3.985 | 15.191 |
|  | 0% | 14.649 | 3.457 | <.001** | 5.357 | 23.941 |
| 60% | 100% | -1.000 | 3.539 | 1.000 | -10.513 | 8.513 |
|  | 10% | 4.603 | 3.541 | 1.000 | -4.914 | 14.120 |
|  | 0% | 13.649 | 3.548 | .001** | 4.114 | 23.184 |
| 10% | 100% | -5.603 | 3.567 | .715 | -15.191 | 3.985 |
|  | 60% | -4.603 | 3.541 | 1.000 | -14.120 | 4.914 |
|  | 0% | 9.046 | 3.550 | .073 | -.496 | 18.588 |
| 0% | 100% | -14.649 | 3.457 | <.001** | -23.941 | -5.357 |
|  | 60% | -13.649 | 3.548 | .001** | -23.184 | -4.114 |
|  | 10% | -9.046 | 3.550 | .073 | -18.588 | .496 |

**References for table 2 and 3 MCID calculations**

1. Sturt JA, Whitlock S, Fox C, Hearnshaw H, Farmer AJ, Wakelin M, et al. Effects of the Diabetes Manual 1:1 structured education in primary care. Diabetic Medicine. 2008;25(6).

2. De Brito GMG, Gois CFL, Zanetti ML, Resende GGS, Silva JRS. Quality of life, knowledge and attitude after educational program for Diabetes. ACTA Paulista de Enfermagem. 2016;29(3).

3. Kellow NJ, Palermo C, Choi TST. Not Scared of Sugar^TM^: Outcomes of a structured type 2 diabetes group education program for Chinese Australians. Health Soc Care Community. 2020;28(6).

4. Reaney M, Zorzo EG, Golay A, Hermanns N, Cleall S, Petzinger U, et al. Impact of conversation map^TM^ education tools versus regular care on diabetes-related knowledge of people with type 2 diabetes: A randomized, controlled study. Diabetes Spectrum. 2013;26(4).

5. Schmitt A, Reimer A, Kulzer B, Haak T, Ehrmann D, Hermanns N. How to assess diabetes distress: comparison of the Problem Areas in Diabetes Scale (PAID) and the Diabetes Distress Scale (DDS). Diabet Med [Internet]. 2016 Jun 1 [cited 2025 Nov 11];33(6):835–43. Available from: https://pubmed.ncbi.nlm.nih.gov/26287511/

6. Polonsky WH, Anderson BJ, Lohrer PA, Welch G, Jacobson AM, Aponte JE, et al. Assessment of diabetes-related distress. Diabetes Care. 1995;18(6).

7. Sigurdardottir AK, Benediktsson R. Reliability and validity of the Icelandic version of the problem area in diabetes (PAID) scale. Int J Nurs Stud [Internet]. 2008 Apr [cited 2025 Nov 11];45(4):526–33. Available from: https://pubmed.ncbi.nlm.nih.gov/17161401/

8. Reddy J, Wilhelm K, Campbell L. Putting PAID to Diabetes-Related Distress: The Potential Utility of the Problem Areas in Diabetes (PAID) Scale in Patients with Diabetes. Psychosomatics [Internet]. 2013 Jan [cited 2025 Nov 11];54(1):44–51. Available from: https://pubmed.ncbi.nlm.nih.gov/23295006/

9. Delahanty LM, Grant RW, Wittenberg E, Bosch JL, Wexler DJ, Cagliero E, et al. Association of diabetes-related emotional distress with diabetes treatment in primary care patients with Type 2 diabetes. Diabetic Medicine [Internet]. 2007 Jan 1 [cited 2025 Nov 11];24(1):48–54. Available from: /doi/pdf/10.1111/j.1464-5491.2007.02028.x

10. Bukhsh A, Khan TM, Phisalprapa P, Duangjai A, Saokaew S, Nawaz MS, et al. Impact of Pharmacist-Led Diabetes Self-Care Education on Patients With Type 2 Diabetes in Pakistan: A Randomized Controlled Trial. Front Pharmacol. 2022;13.

11. Eroglu N, Sabuncu N. The effect of education given to type 2 diabetic individuals on diabetes self-management and self-efficacy: Randomized controlled trial. Prim Care Diabetes. 2021;15(3).

12. Vincze A, Losonczi A, Stauder A. The validity of the diabetes self-management questionnaire (DSMQ) in Hungarian patients with type 2 diabetes. Health Qual Life Outcomes [Internet]. 2020 Dec 1 [cited 2025 Nov 11];18(1). Available from: https://pubmed.ncbi.nlm.nih.gov/33076906/

13. Popoviciu MS, Marin VN, Vesa CM, Stefan SD, Stoica RA, Serafinceanu C, et al. Correlations between Diabetes Mellitus Self-Care Activities and Glycaemic Control in the Adult Population: A Cross-Sectional Study. Healthcare (Basel) [Internet]. 2022 Jan 1 [cited 2025 Nov 11];10(1). Available from: https://pubmed.ncbi.nlm.nih.gov/35052337/

14. Oliveira D, Costa A, Henriques A, Curado MA, Schmitt A, Nogueira P. Validation of the Portuguese version of the diabetes self-management questionnaire-revised (DSMQ-R) in people with type 2 diabetes mellitus. BMC Psychol [Internet]. 2024 Dec 1 [cited 2025 Nov 11];12(1). Available from: https://pubmed.ncbi.nlm.nih.gov/39044279/
